# The COVID-19 Pandemic Impact on the Outcome of Medically Assisted Reproduction Pregnancies

**DOI:** 10.3389/frph.2022.860425

**Published:** 2022-04-25

**Authors:** Mor Huri, Virginia Noferi, Irene Renda, Francesca Piazzini, Silvia Benemei, Maria Elisabetta Coccia

**Affiliations:** ^1^Assisted Reproductive Technology Center, Careggi Hospital, University of Florence, Florence, Italy; ^2^Clinical Trial Unit for Phase 1 Trials, Headache Centre, Careggi University Hospital, Florence, Italy

**Keywords:** COVID-19, pandemic, assisted reproductive technology, reproductive outcomes, early pregnancy loss

## Abstract

**Background:**

The impact of the Coronavirus Disease-2019 (COVID-19) pandemic on pregnancy is not well-understood. During the outbreak, the initial approach suggested by the major societies was to postpone all non-urgent assisted reproductive technology (ART) treatments. Nevertheless, the Italian Society of Fertility and Sterility and Reproductive Medicine considered ethically correct to proceed with ART treatments, as the infertility rate is increasing over time, with a consistent decline in the live birth rate. The objective of our study was to assess the impact of the COVID-19 pandemic on the outcomes of ART pregnancies, in terms of early pregnancy loss, overall success rate, and live birth rate.

**Methods:**

We conducted a single-center retro-prospective cohort study. Patients who underwent ART treatments from 1 March 2020 to 28 February 2021 (pandemic ART cohort, pART; *n* = 749) and from 1 March 2019 to 29 February 2020 (control cohort, CTR; *n* = 844) were enrolled. The study had a duration of 24 months. Patients underwent baseline severe acute respiratory syndrome coronavirus 2 (SARS-Cov-2) nasopharyngeal swab; quantitative serum beta human chorionic gonadotropin (β-hCG) to assess pregnancy; pelvic transvaginal ultrasound; and follow-up until delivery. The study took place at the ART Center of the University Hospital in Florence, Italy.

**Results:**

There were not statistically significant differences on implantation rate (pART 0.348 ± 0.034 vs. CTR 0.365 ± 0.033, CI = 95%, *p* = 0.49), clinical pregnancy rate (pART 0.847 ± 0.044 vs. CTR 0.864 ± 0.038, CI = 95%, *p* = 0.56), and ectopic pregnancy rate (pART 0.008 ± 0.011 vs. CTR 0.01 ± 0.011, CI = 95%, *p* = 0.79). Neither first trimester miscarriage rate was different between the groups (pART 0.224 ± 0.056 vs. CTR 0.213 ± 0.05, CI = 95%, *p* = 0.77) nor second trimester miscarriage rate (pART 0.018 ± 0.018 vs. CTR 0.019 ± 0.017, CI = 95%, *p* = 0.95). Live birth rate remained unchanged during the pandemic (pART 0.22 ± 0.03 vs. CTR 0.239 ± 0.029, CI = 95%, *p* = 0.37) and stable even when compared to our center rate between 2015 and 2019 (pART 0.222 ± 0.03 vs. general rate 0.224 ± 0.014, CI = 95%, *p* = 0.83).

**Conclusion:**

The COVID-19 pandemic did not cause a statistically significant change in the live birth rate and in the pregnancy loss rate. ART during the COVID-19 pandemic can be considered fair and safe, ethically and medically appropriate. Patients and physicians should be reassured that ART pregnancy outcomes do not seem to be jeopardized by the pandemic state.

## Introduction

Coronavirus Disease-2019 (COVID-19) is a respiratory illness caused by the novel severe acute respiratory syndrome coronavirus 2 (SARS-Cov-2). It was first described in December 2019 in Wuhan, China, has since spread globally, and has been declared a pandemic by the World Health Organization (WHO) on 11 March 2020. The COVID-19 outbreak has medical implications that extend beyond the diagnosed patients, resulting in a ripple effect on every aspect of human life and disrupted social and economic function worldwide (1). The emergence of the COVID-19 pandemic has resulted in a global public health emergency. Aiming to enforce social distancing and to preserve hospital resources, many professional societies encouraged the suspension of non-essential medical services. This policy, however, caused a significant reduction in the diagnostic and preventive gynecological procedures, such as hysteroscopy or cancer screening, which may compromise future prognosis (2–4).

This policy affects other fields in women's health, such as the treatment of infertility. During the outbreak, the initial approach suggested by the major societies was to postpone all non-urgent assisted reproductive technology (ART) treatments. On 19 March 2020, the European Society for Human Reproduction and Embryology (ESHRE) suggested preventing the establishment of new pregnancies through deferred embryo transfer, avoiding patients from traveling for fertility treatments, and decreasing additional stress on healthcare systems (5). Similarly, the American Society for Reproductive Medicine (ASRM) suggested that all new treatments and embryo transfers should be suspended, as well as elective surgeries and non-urgent diagnostic procedures (6).

The approach of the Italian Society of Fertility and Sterility and Reproductive Medicine (SIFES-MR) has been different. As infertility is increasing over time, simultaneously with a constant decline in the live birth rate, it was considered ethically correct to allow infertile couples to maintain a viable chance of a future pregnancy (7). In accordance, at the University ART Center in Florence, we proceeded with ART treatments throughout the phases of the pandemic and with the oocyte cryopreservation for oncological patients.

To date, the impact of the COVID-19 pandemic on pregnancy, and specifically ART pregnancies, is not well-understood. In our study, we aimed to assess the impact of the pandemic on pregnancy outcomes in women who underwent ART treatments. To this aim, we compared, in women with positive pregnancy tests, pregnancy outcomes before and after the outbreak of the COVID-19 pandemic and investigated whether there was an adverse effect in terms of pregnancy loss and overall success rate.

## Materials and Methods

We conducted a retro-prospective cohort study at the ART Center of the tertiary maternal and Child Health Careggi University Hospital in Florence, Italy. The study population included subjects who underwent embryo transfer after gonadotropin stimulation (as part of either a fresh cycle or an exogenous hormone-supported frozen embryo transfer) or intrauterine insemination (IUI). In total, 749 subjects were enrolled in the study between March 2020 and February 2021 and were included in the pandemic ART (pART) cohort. As a control (CTR) cohort, we considered all patients (eight-hundreds and forty-four) who were referred to the same center between March 2019 and February 2020.

We included in our study only the *in vitro* fertilization (IVF) cycles in which day-3 embryos or blastocysts were obtained together with adequate endometrial preparation that allowed embryo transfer. The final phase of the fertility treatments was selected to minimize the variability and to create homogeneous groups (as many of the confounding associated with infertility decrease the probability to achieve embryos).

All patients had a quantitative serum beta human chorionic gonadotropin (β-hCG) level performed 11 days after blastocyst transfer or 13 days after day-3 embryo transfer. A pregnancy test was defined as positive if serum β-hCG levels >10 mIU/ml. A pelvic transvaginal ultrasound was performed in our center 2 weeks after a positive pregnancy test.

We established COVID-free protocols for the safety of the patients and the medical personnel.

Before each appointment in our center, all patients were given a triage questionnaire for COVID-19 symptoms and risk factors.

All patients underwent a SARS-Cov-2 nasopharyngeal swab 48 h prior to ovarian pick-up, frozen embryo transfer, or IUI.

If tested positive for COVID-19, the embryo transfer was postponed. Ovarian pick-up, however, was performed with oocyte cryopreservation in case of COVID-19 positivity the same day.

We promoted the COVID-19 vaccination and prioritized the completion of the vaccination cycle before starting the ART treatments.

Pregnancy was documented as a viable clinical pregnancy when an embryo with a fetal heart rate was present. Spontaneous abortion was defined as a non-viable, intrauterine pregnancy with either an empty gestational sac (blighted ovum) or a gestational sac containing an embryo or fetus without fetal heart activity. We distinguished early (within the first 12 + 6 weeks of gestation) and second-trimester spontaneous abortion (between 13 + 0 and 20 + 6 weeks of gestation). Stillbirth was defined as the delivery of a fetus showing no signs of life after 20 + 6 weeks.

A biochemical pregnancy loss was determined in cases of a transient rise in serum β-hCG and was excluded from the clinical pregnancies amount. An ectopic pregnancy was documented in the presence of an extra-uterine gestational sac with a β-hCG level above the threshold for ultrasound visibility, with no evidence of an intrauterine pregnancy.

After the confirmation of the pregnancy viability, an intensive follow-up was conducted until delivery. All pregnancy ultrasound scans, routine prenatal analysis, birth data, and neonatal outcomes were examined. The follow-up was performed remotely to minimize interpersonal interaction. During the follow-up, we also investigated whether the patient tested positive for COVID-19 during the pregnancy.

We therefore compared the live birth rate during the pandemic to our Center overall live birth rate in the previous 5 years (2015–2019).

## Statistical Analysis

The statistical analyses were conducted using the statistical software package JMP version 15.2.0 (SAS Institute Inc., USA). To assess whether the pART cohort and the CTR cohort are homogeneous, we used Fisher's exact test for binomial data and the Mann-Whitney *U*-test for non-parametric data. The data distribution was evaluated using the Shapiro-Wilk test in continuous variables. Continuous variables were represented using mean, standard deviation (SD), and median values, while categorical ones used absolute and relative frequencies and their 95% confidence interval (CI). Categorical data were compared using the χ^2^ test. A *p*-value of ≤ 0.05 was considered statistically significant.

## Results

The pART cohort and the CTR cohort were homogeneous in the variables that have been examined: maternal age, body mass index, type of infertility, and the use of oocytes or sperm donation (Table 1).

**Table 1:** Pandemic assisted reproductive technology (ART) cohort and control cohort characteristics.

	**Pandemic ART cohort**			**Control cohort**			***P*-value**
	**Mean**	**Median**	**SD**	**Mean**	**Median**	**SD**	
Maternal age	38.78	39	4.66	38.47	39	4.99	0.1556
Maternal BMI	22.26	22	2.95	22.66	22	3.46	0.11
Oocyte donation	285/749 (38.05%)			312/844 (36.96%)			0.678
Sperm donation	99/749 (13.2%)			123/844 (14.57%)			0.4688
**Type of infertility**							
Female infertility	294/749 (39.2%)			340/844 (40.3%)			0.772
Male infertility	189/749 (25.2%)			208/844 (24.6%)			
Female and male infertility	107/749 (14.2%)			131/844 (15.5%)			
Unexplained infertility	159/749 (21.2%)			165/844 (19.5%)			
**ART technique**							
Frozen cycle	545/749 (72.7%)			515/844 (61.02%)			<0.001
FIVET/ICSI	195/749 (26.03%)			294/844 (34.83%)			
IUI	9/749 (1.2%)			35/844 (4.14%)			

In the pART cohort, the indications for ART treatment were female infertility (*n* = 294, 39.2%), male infertility (*n* = 189, 25.2%), female and male infertility (*n* = 107, 14.2%), and unexplained infertility (*n* = 159, 21.2%). Similar distribution of the type of infertility was found among CTR cohort (*p* = 0.772, Table 1).

The indications for oocytes donation were poor quality oocytes, iatrogenic infertility, or hypergonadotropic hypogonadism. The indication for sperm donation was azoospermia and cryptorchidism. The percentage of gamete donation remained unchanged with no statistically significant differences (Table 1).

The ART technique distribution, however, was significantly different (*p* < 0.001). Specifically, in the pART cohort, more frozen embryo transfer and fewer fresh IVF cycles have been performed with respect to the CTR cohort (72.7 vs. 61.02% of frozen cycles, Table 1).

The pART cohort included 749 subjects who underwent ART and met the inclusion criteria, 261 (0.348 ± 0.034, CI = 95%) had positive serum β-hCG and 488 patients were failed to conceive. The CTR cohort included 844 subjects, 308 (0.365 ± 0.033, CI = 95%) had positive serum β-hCG and 536 patients were failed to conceive. The statistical differences between the two groups were not found to be significant (*p* = 0.49).

In the pART and CTR cohorts, 40 out of 261 (0.153 ± 0.044, CI = 95%) and 42 out of 308 (0.136 ± 0.039, CI = 95%) subjects with positive serum β-hCG had a biochemical pregnancy, respectively. The comparison between the two groups is not significant, with a *p*-value of 0.56, similar to the comparison on ectopic pregnancies rate (two cases were documented in the pART cohort and three cases in CTR cohort; 0.008 ± 0.011 vs. 0.01 ± 0.011, CI = 95%, respectively, *p*-value = 0.79).

Considering subjects with clinical pregnancy (ectopic pregnancies were excluded), the first trimester miscarriage rate was 0.223 ± 0.056 (CI = 95%; *n* = 49) and 0.212 ± 0.05 (CI = 95%); *n* = 56), in pART and CTR cohorts, respectively, with no statistically significant difference (*p* = 0.77). As for second trimester pregnancy loss, we documented four cases (0.018 ± 0.018, CI = 95%) in pART cohort among clinical pregnancies (two cases due to chromosomal anomaly) and five cases (0.019 ± 0.017, CI = 95%) in the CTR cohort among clinical pregnancies (one case due to chromosomal anomaly), with a *p*- value of 0.95. We also documented one case of stillbirth both in pART cohort and one case in CTR cohort (0.0045 ± 0.009 vs. 0.0038 ± 0.006, CI = 95% among clinical pregnancies, respectively, *p* = 0.9).

In the pART cohort (*n* = 749), we observed 165 (22.02%) deliveries of 176 babies compared to 202 (23.9%) deliveries of 226 babies in the CTR cohort (*n* = 844). The overall live birth rate remained, thus, unchanged during the pandemic with no statistically significant differences (*p* = 0.37). The live birth rate during the pandemic remains unchanged even when compared to our center's general rate between 2015 and 2019 (806 deliveries among 3,601 cycles, pART 0.222 ± 0.03 vs. general rate 0.224 ± 0.014, CI = 95%, *p* = 0.83).

Results are detailed in Table 2.

**Table 2:** The outcome of assisted reproductive technology (ART) pregnancies in the pandemic ART cohort and in the control cohort: results.

	**Pandemic ART cohort**		**Control cohort**		***P*-value**
	***N*°**	**%**	***N*°**	**%**	
**Positive β-hCG test rate**					
Positive β-hCG test	261	34.8	308	36.5	0.49
Negative β-hCG test	488	65.2	536	66.5	
TOTAL ART cycles	749	100.0	844	100.0	
**Biochemical pregnancy rate**					
Biochemical pregnancy	40	15.3	42	13.6	0.56
Positive β-hCG test	261	100.0	308	100.0	
**Ectopic pregnancy rate**					
Ectopic pregnancy	2	0.8	3	1.0	0.79
Positive β-hCG test	261	100.0	308	100.0	
**Early pregnancy loss rate**					
Miscarriage	49	22.4	56	21.3	0.77
Ongoing pregnancy	170	77.6	207	78.7	
Clinical pregnancies	219	100.0	263	100.0	
**Miscarriage < 6 weeks**					
Miscarriage	19	8.7	35	13.3	0.1
Clinical pregnancies	219	100.0	263	100.0	
**Miscarriage 6–12 weeks**					
Miscarriage	30	13.7	21	8.0	0.07
Clinical pregnancies	219	100.0	263	100.0	
**Second trimester loss rate**					
Pregnancy loss	4	1.8	5	1.9	0.95
Clinical pregnancies	219	100	263	100	
**Stillbirth rate**					
Pregnancy loss	1	0.45	1	0.38	0.9
Clinical pregnancies	219	100	263	100	
**Overall live birth rate**					
Live birth	165	22	202	23.9	0.37
TOTAL ART cycles	749	100.0	844	100.0	
**Pregnancy loss rate**					
Overall pregnancy loss	54	24.7	61	23.2	0.79
Clinical pregnancies	219	100.0	263	100.0	

Only six patients from 216 clinical pregnancies (2.78%) were tested positive for SARS-CoV-2 during the pregnancy in the pART cohort. The COVID-19 follow-up results are limited to patients with ongoing pregnancies.

## Discussion

In our cohorts, we studied the impact of the COVID-19 pandemic on pregnancy outcomes among women who underwent ART treatments. A fertility center represents an ideal setting to study the impact of the COVID-19 pandemic on early pregnancy outcomes since all patients underwent predetermination and early follow-up despite the local restrictions.

Many infertile patients decided not to request ART treatments due to the pandemic. During the pandemic, we witnessed a decline in the number of concluded ART cycles as compared to the precedent year (749 vs. 844 cycles). Moreover, the increase in the percentage of frozen embryo transfers with respect to fresh cycles suggests fewer new patients in the pART cohort (Table 1). This decline, despite the unchanged availability of our services, can be attributed to many factors: the local restrictions (national establishment of red areas, preventing people from interregional travels) and the fear about SARS-Cov-2 infection and from potential negative obstetrics outcomes did not encourage physicians and patients to engage in ART. Moreover, the preconceptional analysis and the diagnostic investigations were more difficult to be performed during the pandemic due to the temporary unavailability of some medical services.

It has been suggested that women with COVID-19 have an increased risk of pregnancy loss, which may be attributed to the infection of the placenta by SARS-CoV-2. Recent evidence shows that SARS-Cov-2 binds to angiotensin-converting enzyme 2 (ACE2) receptors to facilitate the fusion of viral and cellular target membranes. ACE2 protein is also expressed in both endometrial epithelial and stromal cells in the proliferative phase of the menstrual cycle and may play a role in the decidualization process. ACE2 was also found in placental syncytiotrophoblast and cytotrophoblast samples from 7 weeks onward. The potential influence of the SARS-Cov-2 infection on early pregnancy outcomes was described in case reports and *in vitro* studies but remains, to date, theoretical (8–12).

The available data, on the other hand, show that SARS-Cov-2 infection is not associated with an increased risk of early pregnancy loss and are in line with our results: an Italian case-control study showed no significant difference in the cumulative incidence of COVID-19 in women who experienced spontaneous abortion than in those with ongoing pregnancy (13). A Danish cohort study found no significantly increased risk of early pregnancy loss in women with SARS-Cov-2 infection and demonstrated that women in their first trimester have not an increased risk of severe COVID-19 disease (14). A Canadian study showed that the outbreak does not seem to affect early first-trimester miscarriage rates in asymptomatic patients (15).

Other potential hazard factors for the outcome of ART pregnancies may be the pandemic state and the impact of social isolation. In the first few months of the pandemic, the community has self-isolated with significant inherent stress, fear, and economic instability (1, 15, 16). Pregnant individuals were experiencing substantially elevated anxiety and depression symptoms during the pandemic, which are found to be related to COVID-19-specific worries, such as the threats to the life of both mother and baby, not getting the necessary prenatal care, and social isolation (17). There is a robust association in literature between psychological stress, emotional wellbeing, and increased rate of miscarriage, irrespective of the mode of conception (18–21). This difference was attributed to the effect of stress on pregnant individuals, mediated by the rise in cortisol, and a possible negative effect on the immune system (15, 22). In accordance, a Chinese study found a correlation between COVID-19-correlated unhealthy lifestyle and poor first-trimester outcome in terms of miscarriages (23).

Nevertheless, our results demonstrate no statistical differences in the pregnancy loss rates between the COVID-19 and pre-COVID-19 periods. We also found no adverse effects in terms of implantation rate, early pregnancy loss, ectopic pregnancy, second-trimester pregnancy loss, or stillbirth. The overall success rate and the live birth rate remain unchanged with respect to the precedent years. It should be considered, therefore, efficacious to proceed with ART treatments during the pandemic.

Other encouraging and interesting data are the very low incidence rates of COVID-19 infection among the pART cohort (only 6 cases among 221 clinical pregnancies), less than the incidence (10.1%) in the general population or among pregnant patients (24, 25). Our patients might be more careful and strictly follow the hygiene norms and social distancing. It is possible that patients with ART, in order to optimize the pregnancy outcomes, tend to create a “Secured-Bubble,” to self-isolate themselves and to avoid potential risks. In accordance, a recent retrospective US study found a very low incidence of SARS-Cov-2 infection among patients with ART (26).

It is important to remember that pregnant women represent a population at high risk, and particular attention is paramount in such cases. Current data from the US Centers for Disease Control and Prevention (CDC) suggest that pregnant women might have an increased risk for severe COVID-19. Among women in reproductive age with COVID-19, pregnant women have an increased risk of hospitalization, intensive-care unit (ICU) admission, and receipt of mechanical ventilation, while mortality rates are similar (27). Furthermore, pregnant patients with COVID-19 have also an increased risk for adverse pregnancy events, such as preterm birth, fetal vascular malperfusion, and premature membrane rupture (28). The SARS-Cov-2 transmission from the mother to the fetus (vertical transmission) remains, however, controversial (29).

The ART treatments should be performed, therefore, in security with restricted COVID-free protocols, prioritizing early vaccination. Recently, it has been suggested that the use of vitamin D and myo-inositol could represent a possible preventive treatment for pregnant patients with ART (30).

In conclusion, we demonstrate that in patients with ART, the COVID-19 pandemic did not cause a statistically significant change in the live birth rate and in the pregnancy loss rate. Even though fewer ART cycles have been completed, the results are encouraging and support the decision to proceed with ART treatments throughout the pandemic.

Our data support that ART during the pandemic is an effective and safe approach, similar to what was observed in a no pandemic period. Since infertility increases over time, and it is difficult to predict the end of the COVID-19 pandemic, infertile patients should ensure access to a viable opportunity to achieve a pregnancy. Patients and physicians should be reassured that pregnancy outcomes do not seem to be jeopardized by the pandemic state.

## Data Availability Statement

The original contributions presented in the study are included in the article/supplementary materials, further inquiries can be directed to the corresponding author.

## Ethics Statement

This study was performed according to the principles of the Declaration of Helsinki (2013) and was approved by the Area Vasta Centro Ethics Committee (code #10189, approval date 05/16/2018 – code #10187, approval date 11/29/2016 – code #18111, approval date 05/20/2021). All data were fully anonymized before being analyzed in a secure database.

## Author Contributions

MH, VN, and IR performed data analysis and wrote the manuscript. MEC conceived the idea, designed the study, and provided a critical review. FP helped with the data analysis. SB provided a critical review. All authors contributed to the article and approved the submitted version.

## Conflict of Interest

The authors declare that the research was conducted in the absence of any commercial or financial relationships that could be construed as a potential conflict of interest.

## Publisher's Note

All claims expressed in this article are solely those of the authors and do not necessarily represent those of their affiliated organizations, or those of the publisher, the editors and the reviewers. Any product that may be evaluated in this article, or claim that may be made by its manufacturer, is not guaranteed or endorsed by the publisher.
